# Parkinson’s disease gene, Synaptojanin1, dysregulates the surface maintenance of the dopamine transporter

**DOI:** 10.21203/rs.3.rs-4021466/v1

**Published:** 2024-03-13

**Authors:** Jacqueline Saenz, Elnaz Khezerlou, Meha Aggarwal, Amina Shaikh, Naga Ganti, Freja Herborg, Ping-Yue Pan

**Affiliations:** 1 Department of Neuroscience and Cell Biology, Rutgers University Robert Wood Johnson Medical School, 675 Hoes Lane West, Piscataway, NJ 08854, USA; 2 Rutgers Graduate School of Biomedical Sciences, Molecular Biosciences Graduate Program, 675 Hoes Lane West, Piscataway, NJ 08854, USA; 3 Department of Neuroscience, University of Copenhagen, Blegdamsvej 3, DK-2200 Copenhagen, Denmark

## Abstract

Missense mutations of PARK20/*SYNJ1* (synaptojanin1/Synj1) have been linked to complex forms of familial parkinsonism, however, the molecular and cellular changes associated with dopaminergic dysfunction remains unknown. We now report fast depletion of evoked dopamine (DA) and altered maintenance of the axonal dopamine transporter (DAT) in the *Synj1+/−* neurons. While Synj1 has been traditionally known to facilitate the endocytosis of synaptic vesicles, we demonstrated that axons of cultured *Synj1+/−* neurons exhibit an increase of total DAT but a reduction of the surface DAT, which could be exacerbated by neuronal activity. We revealed that the loss of surface DAT is specifically associated with the impaired 5’-phosphatase activity of Synj1 and the hyperactive downstream PI(4,5)P_2_-PKCβ pathway. Thus, our findings provided important mechanistic insight for Synj1-regulated DAT trafficking integral to dysfunctional DA signaling in early parkinsonism.

## Introduction

Missense homozygous and compound heterozygous mutations in the PARK20/*SYNJ1* gene are linked to diverse parkinsonism ranging from early onset atypical cases to slow progressing typical Parkinson’s disease (PD) responsive to levodopa [1–8]. Our previous study further demonstrated reduced *SYNJ1* transcripts in a subset of sporadic PD brains [9]. The clinical heterogeneity suggests complex mechanisms underlying *SYNJ1*-associated parkinsonian pathogenesis, which remains poorly understood. Synaptojanin1/Synj1 (encoded by the *synj1* gene) is a lipid phosphatase that contains dual phosphatase activity, via the SAC1-like phosphatase and the 5’-phosphatase domains. Together, they regulate the homeostasis of multiple species of phosphoinositides, including PI(3)P, PI(4)P, PI(3,5)P_2_, PI(4,5)P_2_, etc., which play essential roles in membrane trafficking [10–13]. Complete deletion of Synj1 is perinatally lethal. For almost three decades, Synj1 has been known to facilitate clathrin-mediated endocytosis of synaptic vesicle (SV) [14–19]. In Synj1-null neurons an accumulation of clathrin coated vesicles and pits were observed at the presynaptic terminal [10, 19, 20] and sustained neurotransmission was impaired [18, 21]. Recent studies from us and others further suggested novel roles of Synj1 in autophagy [22–24]. Despite important findings of Synj1 at fast transmitting synapses and glial cells, its role in dysfunctional dopamine (DA) signaling in early parkinsonian pathogenesis remains elusive.

To investigate the impact of Synj1 deficiency in the basal ganglia, our previous work showed that *Synj1* haploinsufficient (*Synj1+/−*) mice exhibited a reduction in striatal DA content and locomotor deficits at 12 months, which was followed by loss of dopaminergic (DAergic) terminals at 18 months [9]. We also demonstrated that synaptic vesicle (SV) recycling in ventral midbrain (MB) neurons is more vulnerable to the *Synj1+/−* mutation than cortical neurons [9, 25]. However, we found that the exocytosis kinetics was not impaired. In parkinsonism-associated *SYNJ1* R258Q mutation knock-in (KI) mice, SV endocytosis was also impaired without affecting exocytosis [26]. More interestingly, large clusters of immunofluorescent dopamine transporter (DAT) were found in the dorsal striatum and these mice exhibit robust locomotor impairment at a young age (2~4 months) [26]. DAT is localized on the periphery of release sites of axonal membranes [27, 28] to reuptake DA and terminate DAergic signaling [27–29]. Somatodendritic DAT also influences membrane excitability via its Na^+^/Cl^−^ symporter function [30, 31]. The finding of abnormal DAT immunofluorescence in the KI mice suggested a possible role of Synj1 in regulating DA signaling via pleiotropic mechanisms other than its well-known role in SV recycling.

DAT recycles like most plasma membrane cargos. However, the molecular mechanisms mediating the recycling/internalization of DAT remain controversial. Both clathrin-dependent and independent endocytosis of DAT have been reported [32–41]. Our previous work suggested that Synj1 may participate in the cocaine-induced DAT trafficking [42], however, whether Synj1 regulates DAT recycling in physiological conditions has not been explored. Neither do we understand the interactions between DA signaling and DAT surface availability in Synj1 deficient conditions. We now use a combination of sophisticated imaging tools including dLight [43, 44] and DAT-pHluorin [42] to dissect the Synj1’s impact on DA homeostasis and the surface maintenance of DAT. Our results revealed an unexpected role of Synj1’s 5’-phosphatase domain in maintaining the surface maintenance of DAT by suppressing the PI(4,5)P_2_-PKCβ pathway, and we provided essential insights for dysregulation of DA signaling via the dynamic availability of DAT.

## Results

### Axonal DA release and clearance were altered in *Synj1+/−* neurons.

**Figure 1.**
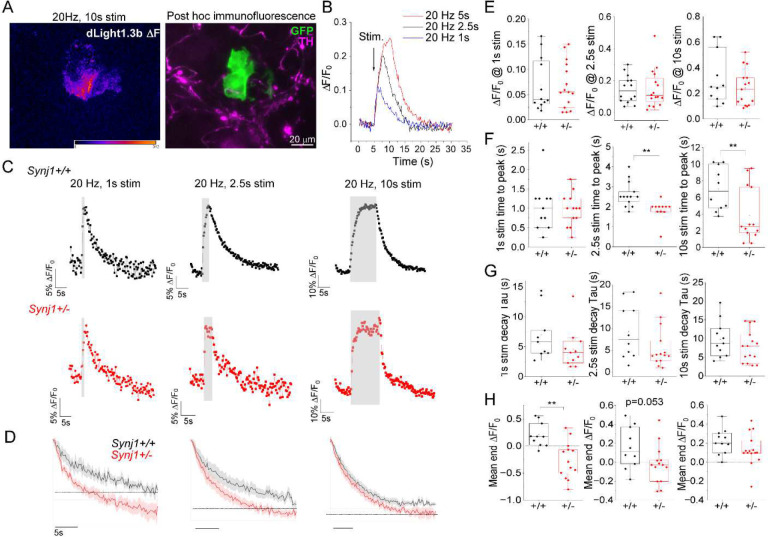
Altered DA release and clearance in the *Synj1+/−* neurons. A, Representative dLight1.3b sniffer cell response to a 20 Hz, 10 sec field stimulation on a heatmap scale (left). *Post hoc* Immunolabeling of tyrosine hydroxylase (TH) and GFP determines the specificity of dLight response from proximal DAergic axons (right). Peripheral DAergic axons do not contribute significantly to the dLight response (data not shown). B, Background subtracted ΔF/F_0_ dLight traces to sequential stimulations at 20 Hz from a representative neuron. C, Representative ΔF/F_0_ dLight traces from *Synj1+/+* and *Synj1+/−* littermate cultures at different stimulation lengths. D, The ΔF/F_0_ dLight responses were normalized to the fluorescence at the end of stimulation and aligned to compare the decay curve (mean ± S.E.M.) relative to baseline (F_0_, dotted line) for *Synj1+/+* and *Synj1+/−* neurons at 20 Hz, 1 sec (left), 2.5 sec (middle) and 10 sec (right) stimulations. E-H, Summarized ΔF/F_0_ peak response (E), time to peak (F), decay time constant (G), and ΔF/F_0_ end fluorescence (H) in *Synj1+/+* and *Synj1+/−* neurons from littermate cultures. **p<0.01, Mann-Whitney test or Student’s *t*-test. Data from 5 batches of cultures.

Our previous study of the *Synj1+/−* mice suggested age-dependent decline of striatal DA metabolism [9]. To further assess how Synj1 regulates DA homeostasis, we sought to measure evoked DA transients using recently developed dLight1.3b DA sniffer cells [43]. Following an initial validation for the dose-responses (Supplemental Fig. 1–1), we generated ventral MB-sniffer cell coculture (see methods, Fig. 1A), which have been shown previously to detect basal and amphetamine-induced DA release [45, 46]. We verified the stimulation-dependent DA release from DAergic axons grown beneath the sniffer cells (Fig. 1A–B), suggesting the sensitivity and reliability of DA sensing. In littermate *Synj1+/+* and *Synj1+/−* cocultures, we measured dLight-DA transients in response to various lengths (1 sec, 2.5 sec and 10 sec) of field electrical stimulation at 20 Hz (Fig. 1C–D). The amplitude of dLight transients was not different between *Synj1+/+* and *Synj1+/−* neurons at all stimulations (Fig. 1E). However, the *Synj1+/−* neurons took significantly shorter time to reach peak response at moderate (2.5 sec) to long (10 sec) stimulations (Fig. 1F). We then evaluated the decline phase of the dLight response, which is indicative of DA clearance. The decay time constant was not significantly altered in *Synj1+/−* neurons (Fig. 1G), however, the dLight fluorescence continued to decline below the baseline following short (1 sec) to moderate (2.5 sec) stimulations (Fig. 1H), suggesting excessive DA clearance in mutant neurons.

### Increased DAT expression in the brains of *Synj1+/−* mice

**Figure 2.**
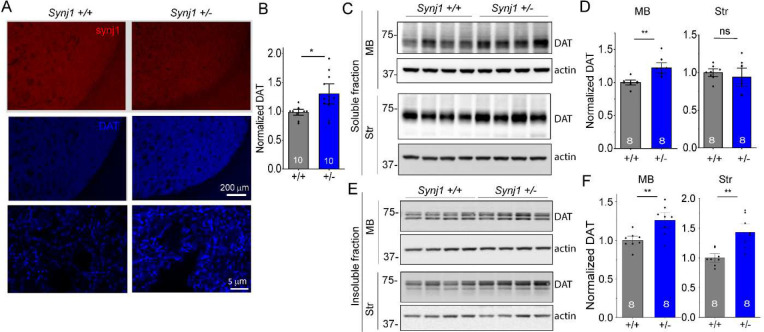
Increased DAT expression in the *Synj1+/−* mouse brains. A, Representative coronal striatal slices from a *Synj1+/+* mouse and a *Synj1+/−* mouse immunolabeled for Synj1 (red) and DAT (blue). Confocal images were captured using a lower and a higher magnification objective and presented in different scales. B, Analysis of the optical density of DAT immunofluorescence at high magnification in the striatum from *Synj1+/+* (n=10) and *Synj1+/−* (n=10) mice at 12–18 months. Each data point is an average value from one mouse. *p=0.024, Student’s *t*-test with Welch correction for unequal variance. C-F, Representative western blots, and densitometry analysis of DAT in the Triton-soluble fractions (C-D) and Triton-insoluble fractions (E-F) of *Synj1+/+* (n=8) and *Synj1+/−* (n=8) male mice. Both the midbrain (MB) and striatum (Str) tissues were analyzed. **p<0.01, Student’s *t*-test.

Recent studies suggested that Synj1 may regulate DAT expression and function [26]. We thus wondered if the altered DA release and clearance dynamics in *Synj1+/−* neurons could be due to changes in DAT, especially as our previous study found lack of evidence for SV exocytosis [25]. We took two approaches to examine the DAT expression in *Synj1+/+* and *Synj1+/−* mice. First, we performed immunofluorescence analysis of DAT in *Synj1+/+* and *Synj1+/−* littermate mice (n=10 in each group) containing both sexes at 12–18 months. An earlier study of the *SYNJ1* R258Q knock-in (KI) mouse found large clusters of immunoreactive structures of DAT in the dorsal but not the ventral striatum [26]. In *Synj1+/−* mice, we did not observe similar DAT clusters in either the dorsal or ventral striatum, however, we observed an overall increase in DAT optical density (Fig. 2A–B). In a separate approach, we performed western blot analysis for *Synj1+/+* and *Synj1+/−* littermate male mice (n=8 in each group) at 10–12 months. Both the MB and striatal tissues were collected, and we examined the DAT level in triton-soluble and triton-insoluble fractions of the sample from each of these brain regions (Fig. 2C–F). A consistent increase of DAT was detected in the *Synj1+/−* MB samples. DAT was not different in the triton soluble fractions of the striatum, but significantly increased in *Synj1+/−* insoluble fractions compared to control.

### *Synj1+/−* neurons exhibit increased total DAT and decreased surface DAT in the axons.

**Figure 3.**
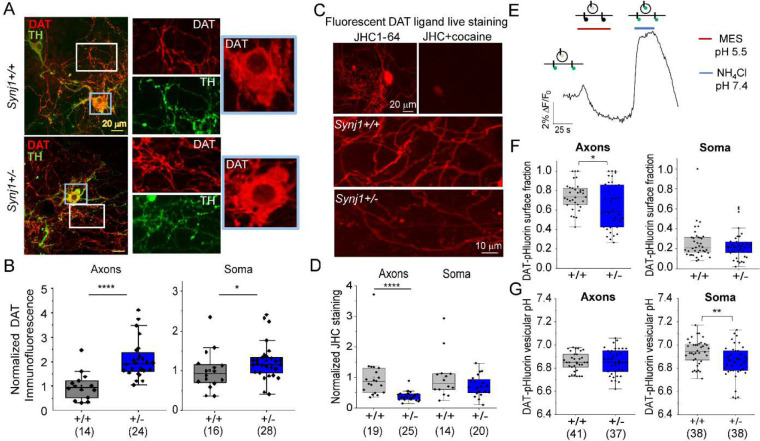
*Synj1+/−* neurons exhibit decreased surface DAT expression. A, Representative immunofluorescence images of cultured ventral midbrain neurons immunolabeled with anti-TH (green) and anti-DAT (red). White and blue boxed regions are axons and soma, respectively, which are magnified on the right panels. B, Normalized immunofluorescence of DAT in the axons (by line selection) and soma (by area selection) of *Synj1*+/+ and *Synj1*+/− DA neurons. *p<0.05, ****p<0.0001. Mann-Whitney tests. Data from 2–3 batches of cultures. C, Top, representative images of MB culture live stained with a fluorescent cocaine analog JHC1–64, which was blocked in the presence of 10 μM cocaine. Bottom, representative images of JHC staining in *Synj1+/+* and *Synj1+/−* cultures. D, Normalized fluorescence of JHC1–64 at the axons (by line selection) and the soma (by area selection). ****p<0.0001. Mann-Whitney test. Data from 2–3 batches of cultures. E, Representative DAT-pHluorin trace to sequential perfusion of a membrane impermeable MES acid solution and a pH7.4 NH_4_Cl solution. Insets, illustration of DAT-pHluorin fluorescence change during the perfusion of the two distinct pH solutions. F-G, Bar graphs summarizing the surface fraction (F) and vesicular pH (G) of DAT-pHluorin in the neuronal axons and soma calculated using the Henderson-Hasselbalch equation. *p<0.05, **p<0.01, Student’s *t-*test.

To better understand DAT expression at the mechanistic level, we examined cultured ventral MB neurons from *Synj1+/+* and *Synj1+/−* mice. Consistent with findings from the brain tissues, total DAT expression at the soma and axons was increased in tyrosine hydroxylase (TH) positive neurons (Fig. 3A, B). To determine if Synj1 regulates surface DAT abundance, we first incubated live MB neurons with a fluorescent cocaine analog, JHC1–64 [47], which has been shown in many recent studies to reveal surface availability of DAT [48, 49]. The specificity of the JHC analysis was verified by the lack of staining in the presence of a DAT blocker, cocaine (Fig. 3C). Live JHC dye staining showed reduced surface DAT (sDAT) in the axons of *Synj1+/−* neurons (Fig. 3C–D) without a significant change at the soma. Second, we expressed a pH sensitive DAT sensor, CAGp-hDAT-pHluorin (Fig. 3E) [42] in cultured ventral MB neurons. Sequential perfusion of a membrane impermeable MES solution (pH 5.5) and a pH neutral NH_4_Cl solution (pH 7.4) (Fig. 3E) allowed us to calculate the surface fraction and vesicular pH of the intracellular DAT-pHluorin using the Henderson-Hasselbalch equation [42, 50–52]. The TH immunoreactivity of the neuron was determined by *post hoc* analysis (raw data in Supplemental Fig. 3–1). Data from both TH+ and TH− neurons were combined in Fig. 3F–G, which indicated that the *Synj1+/−* mutation reduces surface DAT fraction in the neuronal axons (Fig. 3F). The acidity of intracellular DAT vesicles was not different in the axons; however, it was reduced at the soma (Fig. 3G), suggesting their possible degradative fate.

### *Synj1+/−* neurons exhibit impaired DAT trafficking in response to dopamine.

**Figure 4.**
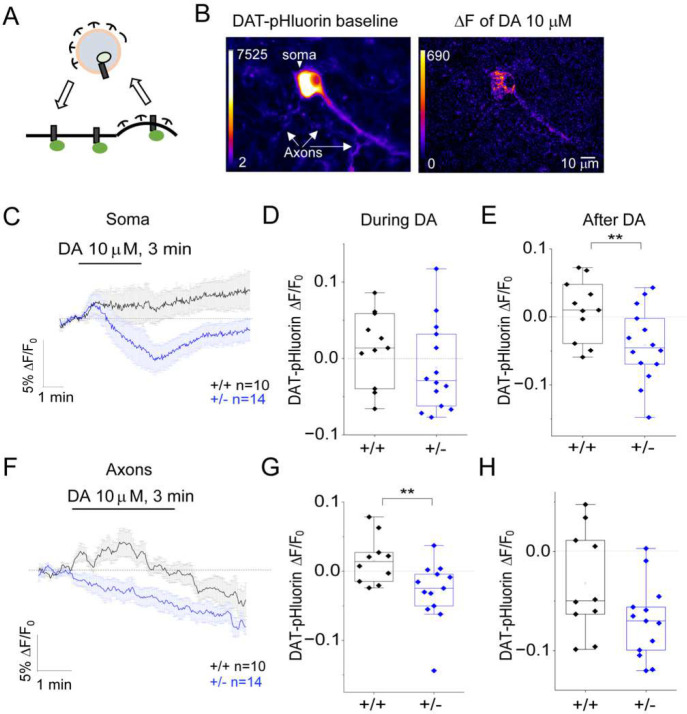
*Synj1+/−* neurons exhibit enhanced DAT internalization in response to DA. A, The working hypothesis of DAT-pHluorin during endocytosis. B, Representative ventral MB neuron expressing DAT-pHluorin at baseline (left) and the fluorescence change (ΔF) in response to perfusion of a 10 μM DA solution (right). The fluorescence was presented using a heat scale. C and F, The average soma (C) and axon (F) DAT-pHluorin response to a 3-minute perfusion of 10 μM DA from *Synj1+/+* and *Synj1+/−* neurons. Data were represented as mean ± S.E.M. D-E, Summary of the averaged soma DAT-pHluorin response during the 3-min DA perfusion (D) and within 1.5 min after the DA perfusion (E). G-H, Summary of the averaged axonal DAT-pHluorin response during the 3-min DA perfusion (G) and within 1.5 min after the DA perfusion (H). **p<0. 01, Student’s *t-*test.

The increased overall DAT expression and reduced sDAT in *Synj1+/−* axons indicated a defect in DAT trafficking at basal conditions. Our previous study using DAT-pHuorin suggested aberrant DAT insertion following cocaine exposure in *Synj1+/−* axons [42]. Nonetheless, cocaine is not a physiological ligand of DAT. To investigate the endogenous mechanisms that may be responsible for intracellular DAT retention, we examined DAT trafficking in response to DA and to membrane depolarization. Cultured midbrain neurons were transfected with DAT-pHluorin to reveal the DAT trafficking dynamics in neurites (Fig. 4A). Perfusion of 10 μM DA hydrochloride in cultured MB neurons led to an average increase in DAT-pHluorin fluorescence to a 3-min DA perfusion (Fig. 4B–D, Supplemental Fig. 4–1A). In contrast, *Synj1+/−* neuronal soma exhibited fluorescence reduction (Fig. 4C–D, Supplemental Fig. 4–1A), and a significant decrease in fluorescence following removal of DA (Fig. 4C, E). An increase in DAT-pHluorin fluorescence was also observed in *Synj1+/+* axons (Fig. 4F–G, Supplemental Fig. 4–1B) during the perfusion of DA followed by a sharp decline, which is likely adaptive responses of DAT to maintain the homeostasis of extracellular DA level. This adaptive trafficking of DAT was not present in *Synj1+/−* axons. DAT-pHluorin exhibited a progressive loss of fluorescence (Fig. 4G–H, Supplemental Fig. 4–1B), indicating DAT internalization.

An earlier study suggested that membrane potential could shape DAT trafficking [53]. We thus wondered if Synj1 deficiency could contribute to synaptic activity-induced loss of DAT. In both the soma and axons of *Synj1+/+* neurons, a 20 Hz, 30 second field stimulation resulted in a reduction of DAT-pHluorin fluorescence (Supplemental Fig. 4–2), consistent with published findings on membrane depolarization induced DAT redistribution [53]. In the soma of *Synj1+/−* neurons, we found a more robust reduction of fluorescence compared to those measured in *Synj1+/+* neurons (Supplemental Fig. 4–2A, C). In the axons, however, we did not observe a significant difference between the *Synj1+/+* and *Synj1+/−* neurons (Supplemental Fig. 4–2B, D).

### The number of large varicosity is not altered in cultured *Synj1+/−* neurons.

**Figure 5.**
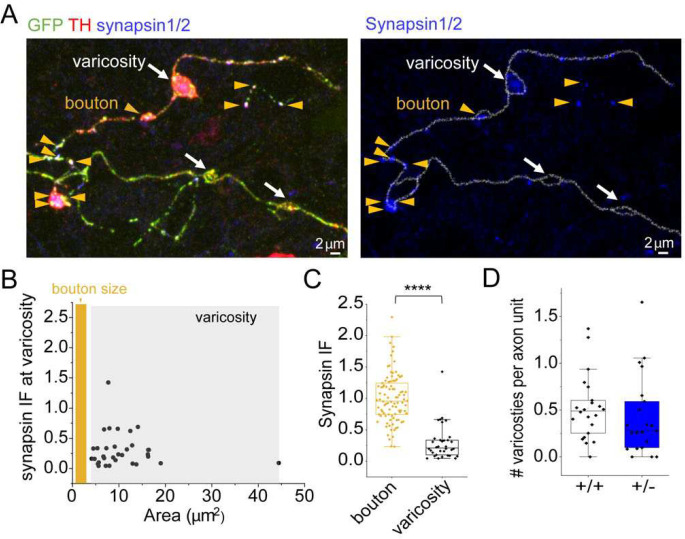
The density of axonal varicosities is not altered in *Synj1+/−* neurons. A, Representative confocal image of cultured WT ventral MB neuron axons immunolabeled with anti-GFP for transfected DAT-pHluorin (green), anti-TH (red) and anti-synapsin1/2 (blue). White arrows point to varicosities and yellow arrow heads point to boutons determined based on size (see B). B, Scattered plot summarizing the size of all varicosities and the synapsin1/2 immunofluorescence of varicosities relative to boutons (see methods). The grey box indicates the size distribution of all varicosities analyzed and the orange box indicates the empirical size of the bouton (<3.14 μm^2^). C, Box plot of synapsin1/2 immunofluorescence of n = 94 boutons and n = 35 varicosities from 3 batches of cultures. ****p<0.0001, Mann-Whitney test. D, Box plot for the number of varicosities/axon area (see methods) in *Synj1+/−* and littermate cultures. *Synj1+/+*: n=22 images, *Synj1+/−*: n=21 images from 3 batches of cultures. p>0.05, Mann-Whitney test.

A striking finding from the *Synj1* R258Q KI mice was the presence of large DAT clusters [26], which was more widespread in a double mutant mouse with simultaneous deletion of PARK19/Auxilin [54, 55]. While we did not observe these clusters in the brain slices of *Synj1+/−* mice, large varicosities are not uncommon in cultured ventral MB neurons. In our previous study, we showed that large varicosities exhibit 25% of sDAT compared to adjacent axons that exhibits 75% in wildtype neurons [42]. We next sought to determine if the reduced axonal sDAT could be due to a higher density of large varicosities in the *Synj1+/−* axons. In cultured *Synj1+/+* and *Synj1+/−* neurons expressing DAT-pHluorin, we immunolabeled with anti-GFP for DAT, anti-TH for DAergic axons and anti-synapsin1/2 for release sites (Fig. 5A). Large varicosities were defined by their size, which segregated from synapsin positive boutons (Fig. 5B, see materials and methods). These large varicosities also exhibited significantly lower and diffused staining for synapsin1/2 (Fig. 5A, C), which distinguished them from boutons. Comparing *Synj1+/+* and *Synj1+/−* axons from 3 batches of cultures, we did not observe any difference for the density of large varicosities (Fig. 5D), suggesting that dystrophic changes in DAT may not be a key pathogenic characteristic of the *Synj1+/−* model.

### Synj1’s 5’-phosphatase domain is essential for the maintenance of sDAT expression.

**Figure 6.**
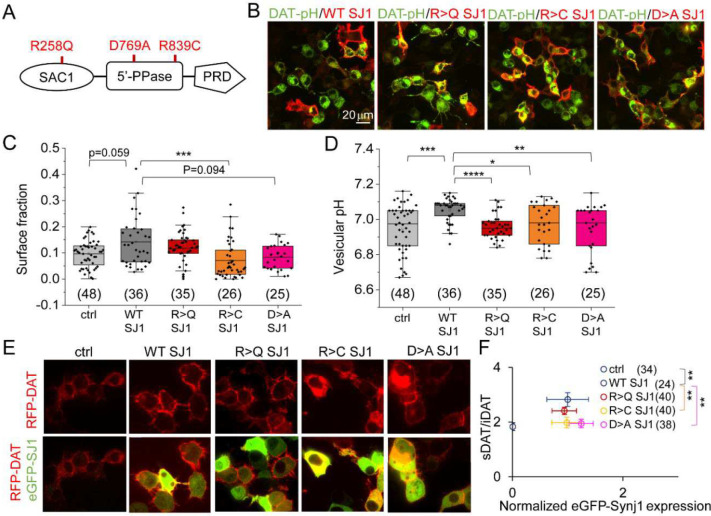
Synj1’s 5’-phosphatase domain is essential for the maintenance of basal level surface DAT. A, Synj1 domain structure and mutations examined in this study. The R258Q and R839C mutations were associated with PD. The D730A mutation completely abolishes the 5’-PPase activity. B, Representative images of N2a cells co-transfected with DAT-pHluorin and different variants of SYNJ1 immunolabeled by anti-GFP (green) and anti-Synj1 (red). C-D, Analysis of DAT surface fraction (C) and vesicular pH (D) for DAT-pHluorin expressing N2a cells. The n=cell number was from 3 independent batches of cultures. E, Representative images of N2a cells transfected with RFP-DAT (red) or co-transfected with RFP-DAT (red) and different variants of eGFP- SYNJ1 (green). F, 2-dimentional distribution of surface RFP/intracellular RFP (sDAT/iDAT) and normalized GFP fluorescence for all cells. n from 2–3 independent batches of cultures. *p<0.05, **p<0.01, ***p<0.001, ****p<0.0001. P values are from Mann-Whitney tests or *Dunn’s post hoc* tests following Kruskal-Wallis ANOVA.

To investigate the Synj1 downstream signaling mechanisms contributing to the reduced sDAT in *Synj1+/−* neurons, we expressed human SYNJ1 cDNAs containing previously characterized functional mutations in the N2a cells to assess the sDAT abundance. The PD mutation, R258Q (R>Q mutant), abolishes the PI(3)P and PI(4)P hydrolysis activities of the SAC1 enzyme without affecting the 5’-phosphase (5’-PPase) activity, whereas, the PD mutation, R839C (R>C mutant), reduces both phosphatases [9]. The non-PD mutation, D769A (D>A mutant), completely abolishes the 5’-PPase activity to hydrolyze PI(4,5)P_2_ [15] and was also included in this analysis (Fig. 6A–B). The MES and NH_4_Cl solutions were perfused as shown earlier (Fig. 3E) to measure the surface fraction of DAT-pHluorin and its vesicular pH. We found that WT SYNJ1 expression led to an increase in sDAT and significantly more alkaline DAT-containing vesicles. Among all mutations, N2a cells expressing the SYNJ1 R>C mutation exhibited the most significant impairment in sDAT fraction and more acidic DAT vesicles compared to WT SYNJ1 expressing cells (Fig. 6C–D). The SYNJ1 D>A mutant expressing cells also exhibited a lower surface fraction and more acidic DAT-vesicles (Fig. 6C–D). We did not observe a significant reduction in the DAT surface fraction in SYNJ1 R>Q expressing cells; however, the DAT-vesicles were significantly more acidic than those in WT SYNJ1 expressing cells (Fig. 6C–D). Taken together, these results suggested that different Synj1 mutations impact DAT trafficking in distinct manners, which could be relevant for genotype-phenotype correlations. Synj1’s 5’-PPase is likely more important for the maintenance of sDAT at basal conditions.

To account for the possibility of differential SYNJ1 expression, we conducted a different set of experiments that co-expressed an N-terminally tagged RFP-DAT with different eGFP-tagged SYNJ1 variants (Fig. 6E). We measured the surface to intracellular DAT (sDAT/iDAT) fluorescence in live N2a cells exhibiting eGFP fluorescence and plotted their sDAT/iDAT values against normalized eGFP-SYNJ1 levels. In agreement with the DAT-pHluorin analysis, while no significant difference was observed for the exogenously expressed SYNJ1 level across all groups, the R>C and D>A mutant expressing cells exhibited significantly lower sDAT/iDAT compared to that from the WT SYNJ1 cells (Fig. 6F). The R>Q mutant was again no different than the WT SYNJ1, further suggesting that the 5’-PPase rather than the SAC1 activity of Synj1 is essential in maintaining the sDAT fraction at the baseline.

### The axonal sDAT is regulated by Synj1 via the PI(4,5)P_2_-PKCβ pathway.

**Figure 7.**
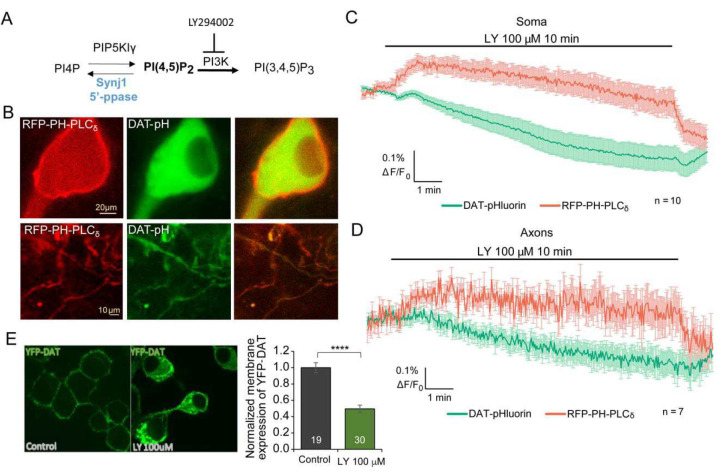
Pharmacologically increasing membrane PI(4,5)P_2_ leads to a reduction of DAT-pHluorin fluorescence in WT neurons. A, Illustration of PI(4,5)P_2_ metabolism regulated by Synj1 and PI3K. B, Representative images of cultured WT MB neuron soma (top) and axons (bottom) co-transfected with DAT-pHluorin and RFP-PH-PLC_δ_. C-D, Dual channel simultaneous imaging of Soma (C) and axonal (D) DAT-pHluorin and RFP-PH-PLC_δ_ fluorescence when treated with a PI3K inhibitor, LY249002 (LY, 100 μM). Data = mean ± S.E.M. n=10 for soma and n=7 for axons from 3 batches of cultures. E, N2a cells expressing YFP-DAT and treated with LY for 10 minutes. Representative images and analysis of relative surface YFP-DAT/ intracellular YFP fluorescence in control and LY treated cells. ****p<0.0001, Student’s *t-*test.

To further investigate the impact of Synj1’s 5’-PPase and its main enzymatic substrate, PI(4,5)P_2_, on DAT trafficking, we sought to transiently increase the plasma membrane PI(4,5)P_2_ and examine the dynamic change of sDAT. In our previous study using antibody labeling of endogenous DAT, we showed that inhibiting phosphoinositide 3-kinases (PI3K) with a pharmacological agent, LY294002 (LY) (Fig. 7A), effectively mimicked Synj1 deficiency by increasing plasma membrane PI(4,5)P_2_ at neuronal soma [9]. To observe the simultaneous change in PI(4,5)P_2_ level and DAT trafficking, we co-expressed a red-shifted PI(4,5)P_2_ biosensor, RFP-PH-PLCδ and DAT-pHluorin in cultured WT ventral MB neurons (Fig. 7B) and performed dual channel live cell imaging. Perfusion of 100 μM LY led to an increase in the RFP fluorescence at both neuronal soma (Fig. 7C) and axons (Fig. 7D) within a minute, suggesting an increase in PI(4,5)P_2_. The DAT-pHluorin fluorescence started to decline with a brief delay following the peak RFP fluorescence, suggesting DAT internalization. Consistently, we observed reduced surface YFP-DAT in N2a cells after a 10-min treatment of 100 μM LY (Fig. 7E), further suggesting that increasing plasma membrane PI(4,5)P_2_ induces DAT internalization.

**Figure 8.**
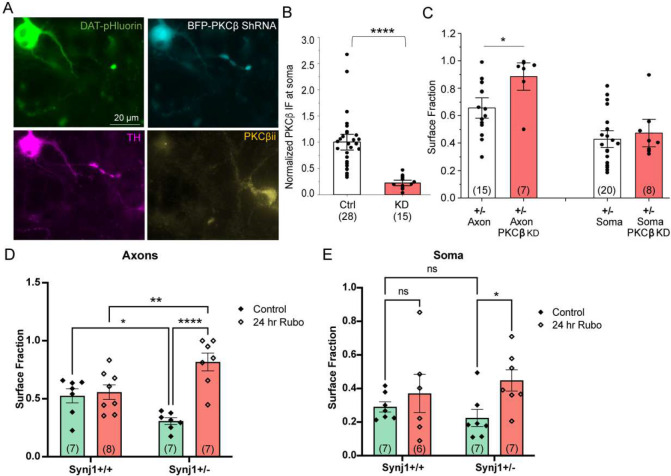
Knocking down PKCβ or inhibiting PKCβ activity restores axonal DAT surface expression in *Synj1+/−* neurons. A, Representative image of a cultured MB neuron co-transfected with DAT-pHluorin and a BFP-tagged shRNA targeting PKCβii. *Post hoc* immunofluorescence for TH and PKCβii. B, Summary of somal PKCβii immunofluorescence in control non-transfected neurons and transfected neurons expressing shRNA. ****p<0.0001, Mann-Whitney test. C, Summary of DAT surface fraction in control *Synj1+/−* neurons and those co-expressing DAT-pHluorin and BFP-shRNA. ****p<0.0001, Mann-Whitney test. D-E, Surface fraction analysis at soma (D) and axons (E) for *Synj1+/+* and *Synj1+/−* neurons in control untreated conditions or following a 24-hour treatment of a PKCβ specific inhibitor, Rubo. *p<0.05, **p<0.01, ***p<0.001, Fisher’s *post hoc* following Two-way ANOVA. n = number of neurons from >=3 batches of cultures.

The above RFP-PH-PLCδ experiment also suggested that increased PI(4,5)P_2_ in *Synj1+/−* neurons could result in the recruitment of phospholipase C (PLC) to produce IP3 and diacyl glycerol (DAG), important co-factors that regulate the activation of a large family of protein kinase C (PKC) [56, 57]. PKC has been shown to phosphorylate DAT and induce its internalization [37, 39, 58–60]. We wondered if Synj1 regulates neuronal DAT maintenance via PKC signaling. Different PKC isoforms may be enriched in specific cell types and even distinct subcellular compartments to be in close proximity to their activators and substrates [57]. Among all PKC isoforms, we specifically examined PKCα and PKCβ, which contains a C2 domain that directly binds PI(4,5)P_2_ [56]. Our immunofluorescence analysis suggested that PKCα expression was only found in 73% of TH+ neuronal soma (out of n=41, from 2 batches of cultures) and majority of TH+ axons were devoid of PKCα staining (Supplemental Fig. 8–1A). Instead, PKCβii immunofluorescence was present in 96% out of n=23 TH+ neuronal soma with evident expression in the TH+ axons as well (Supplemental Fig. 8–1B). We did not observe an increased expression of PKCβii in *Synj1+/−* neurons (Supplemental Fig. 8–1C–D). To assess the possibility of enhanced PKCβii activity in downregulating sDAT in *Synj1+/−* neurons, we transfected DAT-pHluorin with an shRNA targeting mouse PKCβ (Fig. 8A–B). This strategy led to a 75–80% knockdown (KD) of the PKCβii. The sequential perfusion of MES and NH_4_Cl solutions revealed an effective reversal of sDAT fraction in the axons but not at the soma (Fig. 8C). In a parallel study, we treated ventral MB neurons with a PKCβ specific inhibitor, Ruboxistaurin (Rubo, 1 μM), overnight before assessing the sDAT in *Synj1+/+* and *Synj1+/−* neurons. We found that while Rubo did not affect the sDAT in *Synj1+/+* neurons, it effectively reversed sDAT fraction in *Synj1+/−* neurons at both the soma and the axons (Fig. 8D–E). Collectively, we showed that increasing membrane PI(4,5)P_2_ is sufficient to drive DAT internalization in neuronal soma and axons; and the enhanced PKCβ activity is responsible for the Synj1 deficiency-associated loss of axonal sDAT maintenance.

## Discussion

Parkinson’s disease (PD) is often proceeded by altered dopamine (DA) signaling and loss of functional maintenance of DAergic axons [9, 55, 61–70]. In this study, we used the PARK20/*Synj1* haploinsufficient mouse as the model to examine cellular and molecular mechanisms underlying DAergic dysfunction. We employed quantitative imaging approaches to demonstrate that Synj1 deficiency results in altered DA release and clearance kinetics and a trafficking defect for axonal DAT. Our data suggests that the impaired sDAT maintenance could be exacerbated by extracellular DA, and the baseline maintenance of sDAT is regulated through the 5’-PPase of Synj1 and its downstream PI(4,5)P_2_-PKCβii signaling pathway. Thus, our work demonstrates a novel role of Synj1 for presynaptic trafficking and provides mechanistic insight for dysfunctional DAT trafficking, which is integral to DAergic dysfunction in PD.

Patients carrying different *SYNJ1* mutations exhibited heterogeneous clinical manifestations and levodopa responsiveness, suggesting complex pathogenic pathways. Indeed, our results suggests that axonal pathologies associated with *Synj1+/−* mice are dissimilar to the *SYNJ1* R258Q mutation KI mice. For example, large DAT clusters were only reported in the R>Q KI mice [26], but not found in the *Synj1+/−* mice. Further, the *Synj1+/−* mutation elevates plasma membrane PI(4,5)P_2_ [9] and induces DAT internalization, while the R>Q mutation did not impact the surface fraction of the DAT likely due to the unaffected 5’-PPase activity [1, 9]. The PKCβii induced DAT internalization may impact DA signaling and pathogenesis via multiple pathways. First, it could modify the evoked DA transient and lead to altered signaling mediated by phasic DA release. Second, reduced sDAT could serve as a protective mechanism against DA-induced oxidative stress. This perhaps explains the slow progression of the patients with a homozygous mutation in the 5’-PPase domain [7] as well as the *Synj1+/−* mice [9], while the SAC1 mutation carriers exhibit early onset and fast progression of neurodegeneration [1, 3, 4, 26]. Finally, how does the PKC-induced DAT internalization eventually contribute to DAergic decline? We have three hypotheses: (i) The increased basal DA could activate the DA D2 autoreceptor (DRD2) to inhibit DA synthesis and release [71–73]. (ii) Loss of DAT could impair its function as a Na^+^/Cl^−^ symporter and thus reduce the membrane excitability of DA neurons, perhaps with a stronger influence in the nigral pathway [31]. Indeed, loss-of-function mutations of DAT have been reported in patients with parkinsonism [74, 75]. (iii) If the loss of basal sDAT is a result of PKC hyperactivity, we predict a more profound change in many essential membrane transporters, receptors, and ion channels [56, 57]. These trafficking defects could result in a huge burden for the intracellular degradative machineries, culminating in pathogenic inclusions and oxidative stress, characteristic of slow progressing typical PD.

Many earlier studies showed that plasma membrane PI(4,5)P_2_ regulates the function of a multitude of channels and transporters [76]. Direct interaction of PI(4,5)P_2_ with the DAT or serotonin transporter (SERT) has been reported [77–79]. In many of these studies, PI(4,5)P_2_ was suggested as an essential phospholipid in stabilizing transporter functions [77–80]. It is not unlikely that the reduced sDAT in *Synj1+/−* neurons is a compensatory change due to the enhanced uptake activity. While the excessive DA clearance following phasic release (Fig. 1) could be due to sDAT dynamics (Fig. 4F), it could also be a result of enhanced DAT uptake. The precise interaction between DAT trafficking and uptake function requires further investigation.

The most unexpected finding of the study is the role of Synj1 in preventing the endocytosis of the plasma membrane cargo, DAT, which is against our understanding of Synj1 as an endocytic molecule at the presynaptic terminal. The C-terminal proline rich domain of Synj1 is recruited by membrane bending BAR proteins, such as Endophilin and Amphiphysin, during SV endocytosis. Synj1 then cooperates with Auxilin1 (known as PARK19/DNAJC6) [81, 82] to disassemble the clathrin coat for the SV. A recent proteomics study of the *Auxilin−/−* mice identified profound membrane sorting defects implicating DAT as well as SV proteins [55], suggesting that it is perhaps time to revisit the these “endocytic” proteins for their additional roles in clathrin-dependent presynaptic sorting. Several other vacuolar protein sorting receptors, such as VPS35 [83], DNAJC26 (GAK) [84] and DNAJC13 (RME-8) [85] are also implicated in monogenic parkinsonism or PD risk. Whether and how Synj1 interacts with these sorting machineries at the DAergic terminal, and what other essential presynaptic cargos they regulate awaits further investigation. Nonetheless, our study provided first evidence that Synj1 plays pleiotropic roles to dysregulate DA signaling.

In summary, we demonstrate molecular signaling underlying an axonal trafficking defect of DAT in the *Synj1+/−* mice. Our work expands current understanding of the essential gene, Synj1, in regulating neuronal function and provides essential mechanistic insight for DA neuron vulnerability in the early stage of PD.

## Materials and Methods

### Animals

C57BL/6J mice were purchased from the Jax lab. The *Synj1+/−* mouse [10] was a gift from the Pietro De Camilli laboratory at Yale University. *Synj1+/−* mice were crossed to the C57BL/6J mice to generate littermate pups for MB cultures. Mice were housed in the pathogen-free barrier facility at the Rutgers Robert Wood Johnson Medical School Research Tower vivarium. Handling procedures were in accordance with the National Institutes of Health guidelines approved by the Institutional Animal Care and Use Committee (IACUC).

### Cell culture and transfection

Ventral MB cultures [25, 86] were prepared as described previously. Ventral midbrains (containing both VTA and SN) were dissected from P0–1 mouse pups and digested using papain (Worthington, LK003178) in a 34–37°C water bath with gentle stirring and constant oxygenation. MB neurons were then seeded within a cloning cylinder on cover glasses pretreated with Poly-L-ornithine (Sigma, P3655). Cells were plated at a density of 30,000 cells/0.28 cm^2^ and grown in the Neurobasal-A based medium supplemented with GDNF (10 ng/mL, EMD Millipore, GF030). All transfection was performed using Lipofectamine^™^ 2000 (Thermo Fisher, 11668019) on DIV (days in vitro) 5–7 following a company suggested protocol. The DNA-lipofectamine mixture was washed out after 45 min incubation at 37 °C and the growth medium was replaced with a fresh medium supplemented with an antimitotic agent, ARA-C (Sigma-Aldrich, C6645) and Glial cell-derived neurotrophic factor (GDNF, Millipore Sigma, Cat# G1777). Imaging experiments were performed between DIV 13 and DIV 17. N2a cells were cultured and passaged following an ATCC suggested protocol using culture media containing DMEM (Thermo Fisher, 11965118), 10% fetal bovine serum (FBS) (Atlantic Biologicals, S11550), and 5% 10 U/mL Penicillin-Streptomycin (Thermo Fisher, 15140122). Cells were trypsinized using 0.05% Trypsin-EDTA (Gibco, 25300–054) and plated at 30% confluency. Transfection was carried out using Lipofectamine^™^ 3000 (Thermo Fisher, L3000015) following a company suggested protocol the day after plating. The transfection mixture was left in the medium until the day of imaging (typically within 24–48 hours).

### Constructs

The CAGp-hDAT-pHluorin was engineered and validated as reported in our previous study [42]. The pHluorin was inserted in the second extracellular loop of human DAT. The tagRFP-C1-DAT-HA was from Addgene (#90265). The pEGFP-C1-FLAG-WT hSYNJ1–145 construct was originally gifted by Dr. Pietro De Camilli at Yale University. The pEGFP-C1-FLAG-R258Q hSYNJ1–145, pEGFP-C1-FLAG-R839C hSYNJ1–145, and pEGFP-C1-FLAG-D769A hSYNJ1–145 as well as their EGFP-deleted counterparts were generated via site-directed mutagenesis (Agilent Technology QuickChange 200517) and reported in our previous studies [1, 23, 25]. The PKCβ shRNA (pRP[shRNA]-TagBFP2-U6>mPrkcb) was constructed by vector builder with a BFP tag and hairpin structure targeting mouse PKCβ: GAGATTCAGCCACCTTATAAA.

### dLight1.3b sniffer cell co-culture

Dlight1.3 sniffer cells were thawed and prepared as previously described [43]. Cells were maintained in a selection medium containing 90% DMEM (ThermoFisher #11965092), 10% fetal bovine serum (Atlanta Biologicals #S11550H), 1% Pen/Strep (ThermoFisher #15140122), 15 μg/mL Blasticidin (Millipore Sigma, Cat# 15205) + 200 μg/mL Hygromycin (Millipore Sigma, Cat# H3274). For co-culture with the ventral MB neurons, an 8 × 8 mm cloning cylinder were placed on the MB culture at DIV 13 and filled with neuronal medium. Sniffer cells were seeded at 20,000 / cylinder after trypsinization. Doxycycline (1 μg/mL) were added to the co-culture the next day for 24 hours and imaging experiments were performed on DIV 15. Perfusion of different concentrations of DA solutions was carried out similarly as described in the *DAT-pHluorin imaging* section. Field electrical stimulation was delivered in a time frame locked manner via a custom-built stimulation chamber with two platinum electrodes at 10 V/cm by the A310 Accupulser and A385 stimulus isolator (World Precision Instruments) [86, 87]. A 1 ms pulse was used to evoke single action potentials. Images were acquired at 4 Hz.

### JHC dye live staining

Staining was performed following previously published protocols [48, 49]. Briefly, MB cultures were washed three times in cold JHC-buffer containing: 25 mM HEPES, pH 7.4, with 130 mM NaCl, 5.4 mM KCl, 1.2 mM CaCl_2_, 1.2 mM MgSO_4_, 5 mM D-glucose. Cell culture was then incubated with 50 nM JHC1–64 dye diluted in JHC buffer containing 1 mM L-ascorbic acid, at 4°C to avoid internalization for 30 minutes. For negative control, 10 μM cocaine was included in the JHC buffer. After incubation the dye was washed away three times with ice cold JHC- buffer followed by immediate fixation with 4% PFA at room temperature. Images were taken immediately using confocal microscopy.

### DAT-pHluorin live imaging

For live cell imaging, cells on cover glass were mounted on a custom-made laminar-flow chamber with constant gravity perfusion (at a rate of ~0.2–0.3 mL/min) of a Tyrode’s salt solution containing 119 mM NaCl, 2.5 mM KCl, 2 mM CaCl_2_, 2 mM MgCl_2_, 25 mM HEPES, 30 mM Glucose, 10 μM 6-cyano-7- nitroquinoxaline-2,3-dione (CNQX), and 50 μM D, L-2-amino-5-phosphonovaleric acid (AP-5) and buffered to pH 7.40. The NH_4_Cl medium contains: 50 mM NH_4_Cl, 70 mM NaCl, 2.5 mM KCl, 2 mM CaCl_2_, 2 mM MgCl_2_, 25 mM HEPES, 30 mM Glucose, 10 μM CNQX, and 50 μM AP-5, buffered to pH 7.40. The MES medium contains: 25 mM MES, 70 mM NaCl, 2.5 mM KCl, 2 mM CaCl_2_, 2 mM MgCl_2_, 30 mM Glucose, 10 μM CNQX, and 50 μM AP-5, buffered to pH 5.50. Perfusion of the Tyrode’s solution as well as pharmacological reagents (DA hydrochloride and LY294002) buffered in Tyrode’s solution is regulated by Valvelink 8.2 and the NH_4_Cl or MES solutions were perfused by pipettes. All chemicals were purchased from Sigma-Aldrich or Tocris Bioscience unless otherwise noted. For the overnight Ruboxistaurin treatment study, Ventral MB cultures transfected with DAT-pHluorin were treated with 1μM Ruboxistaurin (Rubo, LY333531) HCl (Selleck Chemicals, Cat #S7663) diluted in the culture media on DIV 15–16 for at least 18 hours not to exceed 24 hours prior to live imaging using MES-NH_4_Cl to measure the surface fraction of DAT. Temperature was clamped at ~31.0 °C at the objective throughout the experiment. Images were acquired using a highly sensitive, back-illuminated EM-CCD camera (iXon+ Model DU-897E-BV, Andor Corp., CT, USA). Nikon Ti-2 wide-field microscope is modified with Spectra-X (Lumencor) as the light source for shuttered illumination. pHluorin fluorescence excitation and collection were using a Nikon PLan APO 60X 1.40 NA objective using 525/50m emission filter and 495LP dichroic filters (Chroma, 49002).

### Western blots

Brain samples were lysed using a Triton-based lysis buffer containing 50 mM Tris-HCl (pH 7.5), 150 mM NaCl, 1% Triton as well as protease and phosphatase inhibitors as previously described [9, 23, 25]. After centrifugation at 16,000 g, 4°C for 30 min, supernatant was collected for protein quantification using the Pierce BCA assay (Thermo 23227). Typically, 10–20 μg of total proteins were loaded for each sample on the Invitrogen 4–12% Bis-Tris gel and transferred to a PVDF membrane (Thermo 88518).

### Immunohistochemistry

Mice were anesthetized using the isoflurane drop method and perfused transcardially with 4% fresh paraformaldehyde, and post-fixed with 4% paraformaldehyde for over two hours. Dissected brains were cryoprotected in 30% sucrose prior to flash-freezing in the OCT-compound media (SAKURA). Coronal sections were sliced at 40 μm thickness on a Leica CM 3050 S research cryostat and kept at an anti-freeze medium for immunohistolochemical (IHC) analysis. IHC was carried out following a standard protocol as previously described [9]. Briefly, tissue slices were washed in 1X PBS and blocked in 5% goat serum for 30–60 minutes. Primary antibodies diluted in 5% goat serum were applied and incubated overnight at 4°C, followed by Alexa Fluor^®^ secondary antibodies (Invitrogen^™^). The tissue slices were then subjected to extended washing using 1X PBS to reduce background fluorescence before mounting with Diamond Antifade Mountant (Thermo Fisher Scientific, P36962). Immunofluorescence was analyzed using a Nikon CREST spinning disk confocal microscope. 3–5 regions per brain slice were selected randomly for imaging, and 2–3 slices per mouse were used for analysis. The same imaging parameters were set for each batch of culture. Image stacks were taken at different focal planes at 0.9 μm interval and a maximum projection image was generated for each stack via ImageJ for analysis.

### Antibodies

The following primary antibodies were used for IHC: rat anti-DAT (EMD Millipore, MAB369, 1:1000 dilution), rabbit anti-synj1 (Novus Biologicals, NBP1–87842, 1:500 dilution). Antibodies used for western blots: rabbit anti-DAT (Millipore-Sigma, AB2231, 1:1000), mouse anti-DAT (ThermoFisher, MA524796, 1:1000), mouse anti-actin (Cell Signaling, 3700, 1:1000). Antibodies used for immunocytochemistry: rat anti-DAT (EMD Millipore, MAB369, 1:1000 dilution), guinea pig anti-synapsin 1/2 (Synaptic System, 106004, 1:500 dilution), chicken anti-GFP (ThermoFisher, A-10262, 1:1000), rabbit anti-TH (Novus Biologicals, NB300–109, 1:1000), mouse anti-TH (Sigma, T2928, 1:1000), rabbit anti-synj1 (Novus Biologicals, NBP1–87842, 1:500), mouse anti-PKCα (Novus Biologicals, NB600–201SS, 1:1000), mouse anti-PKCβii(F-7) (Santa Cruz, sc-13149, 1:50).

### Varicosity analysis

Confocal stacks of fixed and immunolabeled cells were captured at 0.5 μm steps and projected by maximum intensity. Varicosity size was determined by tracing large TH+ structures using the freehand tool in ImageJ. Bouton size was determined by placing 2 × 2 μm circular regions of interests (ROIs) using the Time Series Analyzer plugin. All synapsin1/2 immunolabeled puncta in our analysis can be placed within the 2 × 2 μm circular ROIs, therefore, we empirically determined the bouton size to be 0–3.14 μm^2^. The total axon area for each image was determined by generating a binary image for synapsin1/2 immunofluorescence channel. The total pixel area for synapsin1/2 was used as a proxy for axon area to determine the density of large varicosities in the imaging field.

### Data analysis and statistics

DAT-pHluorin surface fraction was calculated as detailed in our previous publication [42]. All images were analyzed using ImageJ or FIJI. Measurements of axon or surface fluorescence were obtained using the segmented line function. Measurements of soma or intracellular fluorescence were obtained using the freehand selections. All data was subject to a normality test. Student’s *t-*test, One-way ANOVA or Two-way ANOVA was used only if all datasets were normally distributed. If one or more datasets did not conform to normal distribution, Mann-Whitney test or Kruskal-Wallis ANOVA was used. All western blots had over 2 technical repeats and all cell culture analyses were from 2–6 independent cultures.

## Supplementary Material

Supplement 1
