# Supplementary material for: Parkinson’s disease gene, Synaptojanin1, dysregulates the surface maintenance of the dopamine transporter
Source: Res Sq. 2024 Mar 13:rs.3.rs-4021466. Preprint. [Version 1] doi: 10.21203/rs.3.rs-4021466/v1 (PMC10980101; doi:10.21203/rs.3.rs-4021466/v1)
Supplement: Supplement 1 [file NIHPPrs4021466v1-supplement-1.pdf]

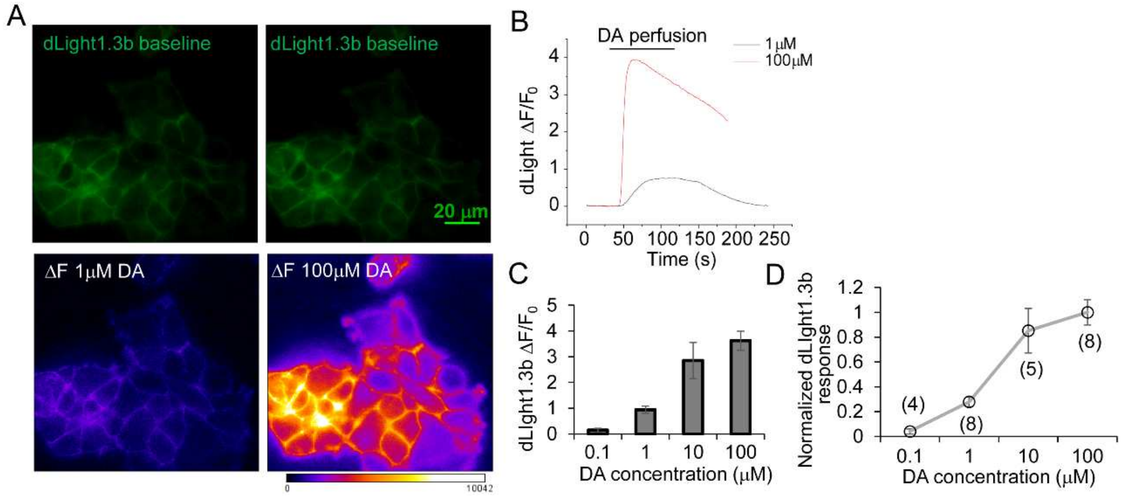

Supplemental Fig. 1-1. Validation of dLight1.3 sniffer cell dose response

A, Representative sniffer cells at baseline (top) and their ΔF responses when perfused with 1 μM (bottom left) or 100 μM (bottom right) DA. B, Background subtracted dLight $\Delta F/F_0$ responses to the corresponding perfusion. C-D, Dose-dependent dLight responses in raw $\Delta F/F_0$ values (C) or when normalized to the max response at 100 μM (D). n = number of experiments.

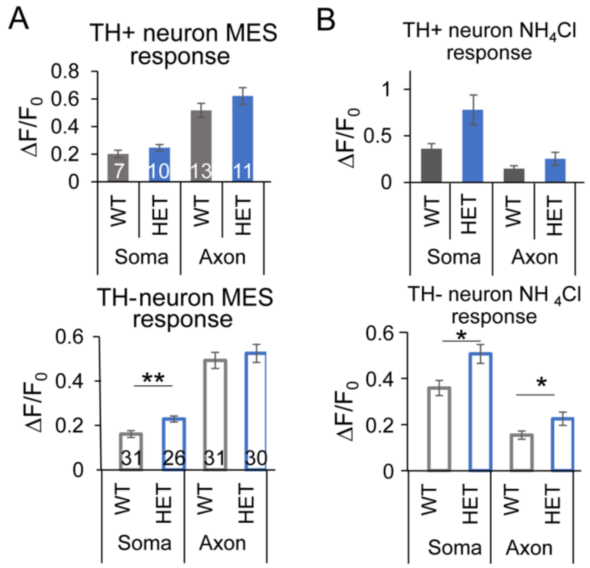

Supplemental Fig. 3-1. Raw measurements of MES and NH_4Cl responses

A-B, Bar graphs summarizing DAT-pHluorin MES (A) and NH_4Cl (B) responses at various compartments of TH+ and TH- midbrain neurons. P values are from Tukey's *post hoc* following one-way ANOVA. *p<0.05; **p<0.01.

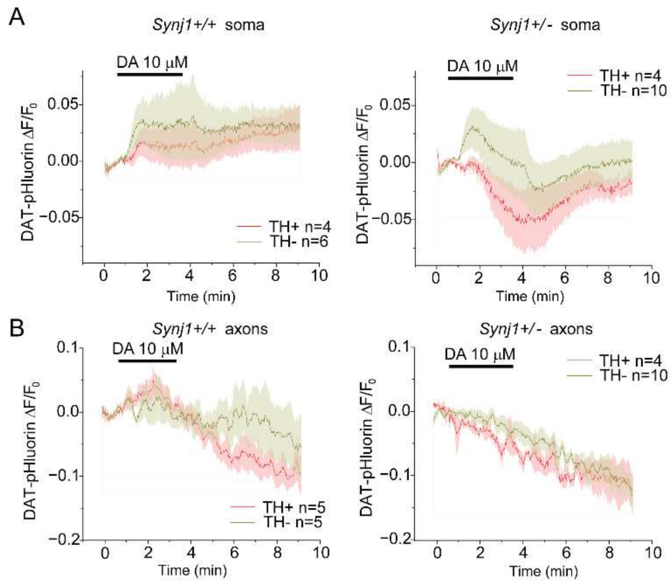

Supplemental Fig. 4-1. DA-induced DAT-pHluorin responses in TH+ and TH- neurons.

Post hoc analysis of the data from Figure 4 showing averaged responses in TH+ and TH- neuronal soma (A) and axons (B) in response to DA perfusion. Data = mean ± S.E.M.

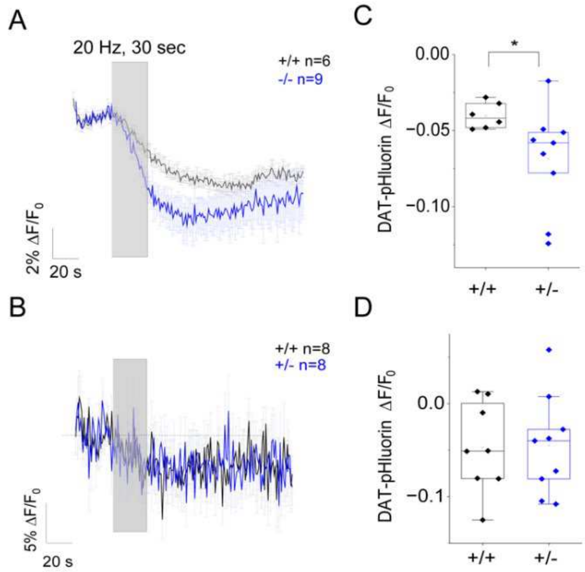

Supplemental Fig. 4-2. DAT-pHluorin response to neuronal action potentials.

A-B, The average soma (A) and axon (B) DAT-pHluorin response to 20 Hz, 30 sec electrical stimulation. Data = mean ± S.E.M. C-D, Summary of the averaged peak soma (C) and axon (D) DAT-pHluorin response during the stimulation. *p<0.05, Student's *t*-test.

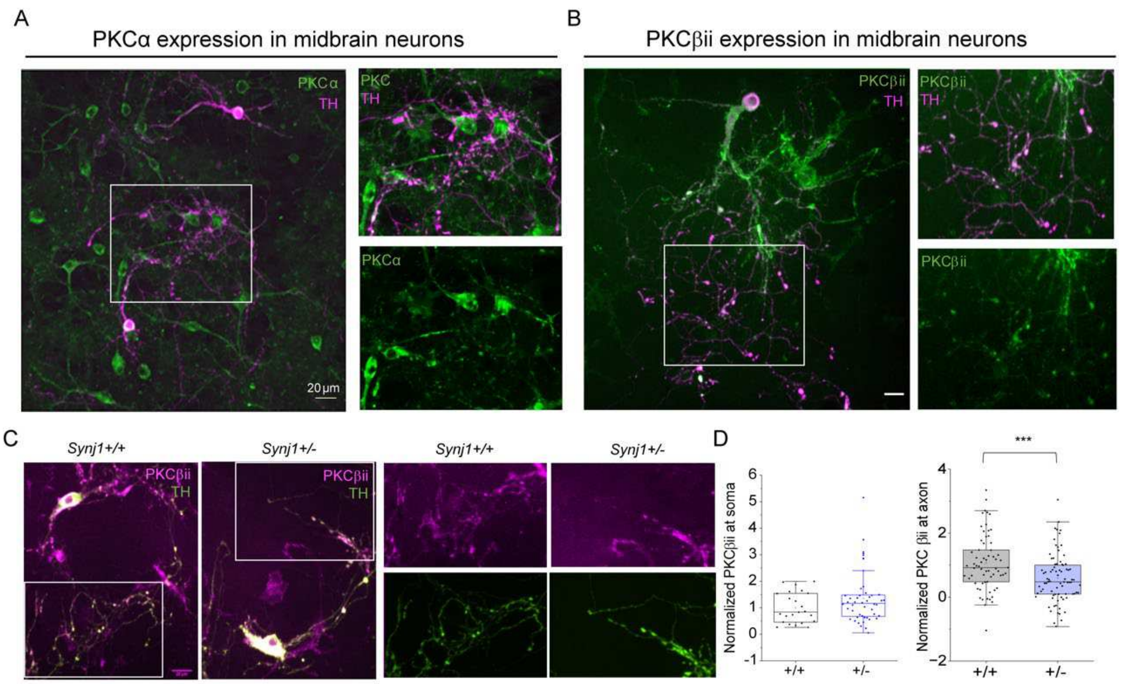

Supplemental Fig. 8-1. PKCβii is expressed in DA neurons and their axons.

A-B, Representative images of a cultured MB neurons co-immunolabeled with anti-PKCα (A) or anti-PKCβii (B) with anti-TH. C, Representative images of a *Synj1+/+* and *Synj1+/-* MB neurons co-immunolabeled with anti-PKCβii and anti-TH. D, Analysis of normalized soma (left) and axonal (right) PKCβii immunofluorescence. $p=0.251$ for soma comparison, Mann-Whitney test, and $p=5.855e-4$ for axon comparison, Student's *t*-test.
